# Pathology of wild-type and toxin-independent *Bacillus anthracis* meningitis in rabbits

**DOI:** 10.1371/journal.pone.0186613

**Published:** 2017-10-31

**Authors:** Assa Sittner, Elad Bar-David, Itai Glinert, Amir Ben-Shmuel, Shay Weiss, Josef Schlomovitz, David Kobiler, Haim Levy

**Affiliations:** Department of Infectious Diseases, Israel Institute for Biological Research, Ness Ziona, Israel; Hungarian Academy of Sciences, HUNGARY

## Abstract

Hemorrhagic meningitis is considered a complication of anthrax and was reported in about 50% of deadly cases in humans and non-human primates (NHP). Recently we demonstrated in Guinea pigs and rabbits that 100% of the *B*. *anthracis*-infected animals presented histopathology of meningitis at the time of death, some without any sign of hemorrhage. A similar pathology was observed in animals that succumbed following infection with the toxin deficient mutant, thus indicating that anthrax meningitis is a toxin-independent phenomenon. In this manuscript we describe a histopathological study of the *B*. *anthracis* infection of the central nervous system (CNS). Though we could find sporadic growth of the bacteria around blood vessels in the cortex, we report that the main infiltration route is the choroid plexus. We found massive destruction of entire sections of the choroid plexus coupled with massive aggregation of bacilli in the ventricles, in close proximity to the parenchyma. The choroid plexus also contained significant amounts of intravascular bacterial aggregates, often enclosed in what appear to be fibrin-like clots. The high concentration of these aggregates in areas of significant tissue destruction combined with the fact that capsular *B*. *anthracis* bacteria have a low tendency to adhere to endothelial cells, might suggest that these clots are used as an adherence mechanism by the bacteria. The major histopathological finding is meningitis. We find massive bacterial growth in the meninges without evidence of encephalitis, even when the bacteria emerge from a parenchymal blood vessel. Erythrocytes were present within the meningeal space but no clear vasculitis could be detected. Histology of the brain stem indicates meningitis, edema and hemorrhages that might explain death from suffocation due to direct damage to the respiratory center. All of these processes are toxin-independent, since they were observed following infection with either the wild type strain or the toxin-deficient mutant. Herein, we propose that the first step of anthrax-meningitis is bacterial adhesion to the blood vessels by manipulating coagulation, mainly in the choroid plexus. The trapped bacteria then destroy sections of the choroid plexus, resulting in penetration into the CSF, leading to meningitis and hemorrhage. Death could be the result of increased intracranial pressure and/or damage to the brain stem.

## Introduction

Systemic anthrax is the result of infection with the gram positive spore forming bacterium *Bacillus anthracis*. The spores can infect the host via three major routes [1, 2]; skin lesions, digestion of contaminated food or inhalation. Skin infection usually results in typical coal-like wounds that in the absence of treatment can progress in about 30% of the cases into systemic disease and death. Digestion of inadequately cooked meat from sick animals results in a gastro-intestinal infection that, if untreated, develops into a lethal systemic disease. Inhalation anthrax is caused by inhaling spores that consequently undergo uptake by macrophage and/or dendritic cells, which migrate to the proximal lymph node. The transferred spore germinates, producing the anti-phagocytic capsule [3] and the lethal- and edema toxins [4] thereby initiating a deadly systemic disease, probably by disseminating through the lymph system to the spleen and to the blood stream.

In the systemic phase of anthrax, vegetative bacteria disseminate via the bloodstream throughout the host body. This dissemination results in accumulation of the pathogen in various tissues, with evident histological damages, followed by death of the host. We demonstrated that artificial systemic disease induced by intravenous (IV) injection of encapsulated vegetative bacteria results in organ dissemination and death, mimicking inhalation anthrax (the result of intranasal (IN) instillation). The major difference between the IN and IV inoculation was the role of the toxins in the development of the deadly infections. As expected, the toxins are crucial for spore based IN infection. Nevertheless, once the bacteria are blood borne, the toxins become redundant and the host will succumb to the infection even in their absence. There are two major differences between the diseases initiated by the IN versus IV inoculation of the Vollum strain: First, the time to death is significantly shorter following IV inoculation, with animals succumbing within 24 h [5] compared to 48 to 72 h post infection for IN instillation [6]. Second, the bacteremia levels at death are lower in the IV injected rabbits (10^6^−10^7^ CFU/ml) [5] compared to the IN inoculated (10^8^−10^9^ CFU/ml) [7], probably reflecting the difference in time to death. Quantification of the bacterial burdens in various organs shows a tendency towards significant bacterial accumulation in the brain following IV inoculation [5].

In humans we can distinguish three phases in the development of anthrax: an asymptomatic incubation period of 1–6 days, a flu-like stage with nonspecific symptoms that can last 1–5 days and finally, a short acute stage of 1–2 days resulting in the death of the patient [8, 9]. These three stages are not identifiable in animal models (i.e. guinea pigs, rabbits and non-human primates (NHP)), and other than a transient increase in body temperature, the only clinical sign of disease is acute respiratory distress shortly before death. The onset of bacteremia (appearance of bacteria in the blood stream) defines the transition between the incubation period and the initiation of systemic disease [10]. This distinction was used to study the efficacy of antibiotic treatments to prevent or cure the disease [7]. While post exposure prophylaxis is generally highly effective, initiation of treatment during the late systemic phase is not and has a very high failure rate. Previously we demonstrated in the rabbit model that the efficacy of antibiotic treatment depends on the bacteremia levels, as most of the first line antibiotics (i.e. Ciprofloxacin, Doxycycline, Amoxicillin/Clavulonic acid) cure the vast majority of the animals with bacteremia of up to 1x10^5^ CFU/ml [7, 11]. Treatment efficacy drops in animals with bacteremia above this level and up to 1x10^6^ CFU/ml, completely failing in animals with bacteremia levels higher than that. One of the treatment challenges at the acute stage is the onset of meningitis in more than 50% of the cases in humans and NHP, which manifests as massive hemorrhages in the brain cortex (cardinal's cap) [12, 13]. Patient data reported between 1945 to 2014 [14] demonstrates that antibiotic treatment of patients that were negative for meningitis was effective in 71% of the cases while the treatment of meningitis-positive patients was effective in less than 20%. In the rabbit model, careful analysis of brain bacterial loads in correlation with different bacteremia levels revealed that specific accumulation of bacteria in the brain starts at bacteremia levels higher than 10^4^ CFU/ml [15], a finding that correlates well with the reduction in the efficacy of the antibiotic treatment and the development of meningitis in humans. Furthermore, recent CDC guidelines for the treatment of anthrax patients [16] distinguish between three groups; post exposure prophylaxis, cases where meningitis was ruled out and cases with suspected meningitis [16]. Our finding that rabbits that succumbed to systemic anthrax exhibited meningitis pathology [5, 15] strengthens our use of this model to study the development of anthrax–meningitis.

Bacterial crossing of the brain barriers has been described as occurring through three major pathways [17, 18]; 1. Specific translocation through the blood vessels' tight-junctions, 2. Breach of the blood vessels of the meninges and 3. Translocation through the relatively permeable blood vessels of the choroid plexus (CP). The CP is a tissue rich in blood vessels, responsible for the transfer of metabolites between the blood and cerebral-spinal fluid (CSF) which also serves as an entry point for several CNS pathogens. CSF formed in the choroid plexi flows through the cerebral ventricles and the subarachnoid space to its ultimate sites of reabsorption into the blood stream via the arachnoid villi of the dural sinuses. A substantial portion of subarachnoid CSF cycles through the brain interstitial space, entering the parenchyma along paravascular spaces that surround penetrating arteries and clearing along paravenous drainage pathways.

The pathology of anthrax in animal models and human was previously described, referring to the brain damage as a consequential effect. These reports describe meningitis in mice [19, 20], Guinea pigs [21], rabbits [22], NHP [12, 23] and humans [13], as part of the general pathology. The main parameter scored was brain hemorrhage. In the victims of the Sverdlovsk accidental spore release, 90% of the cases showed brain hemorrhage. Analysis in NHP experiments demonstrates hemorrhage in about 50% of fatal anthrax cases. This phenomenon is relatively rare in other animal models. In rabbits, for example, hemorrhage was detected only in about 20% of the cases. Previously we demonstrated high bacterial loads in the brains of Guinea pigs and rabbits [5, 15]. In addition, we and others have documented meningitis in these animal models [22]. Although toxins were assumed to play a major role in this process, we demonstrated that in these animal models, infections with bacteria lacking the toxins resulted in similar gross pathology [6, 15, 24]. In this manuscript we attempt to elucidate the mechanism by which *B*. *anthracis* invades the BBB, using CNS histopathology of animal models that succumbed to systemic anthrax. We examine the pathology of anthrax meningitis in the rabbit model, focusing on the three crucial steps of bacterial brain invasion: adherence, penetration and proliferation. We propose that the bacteria take advantage of blood-clots to adhere to blood vessels. Following this adherence, the bacteria secrete factors that influence endothelial permeability and breach the blood vessel, probably due to a combined effect of the increased blood pressure resulting from the blood clots. We propose the choroid plexus is the target site for brain penetration. Our findings indicate that anthrax causes meningitis but not encephalitis and the major target of this meningitis is the cerebellum. We speculate that death is the result of increased intra-cranial pressure that affects the brain stem.

## Material and methods

### Bacterial strains, media and growth conditions

*B*. *anthracis* strains used in this study are Vollum (ATCC 15578) and Vollum*ΔpagΔlefΔcya*, (Toxin deficient Vollum strain) a complete deletion of the *pag*, *lef* and *cya* genes [6]. Sporulation was carried out using G broth, as previously described [25].

### Infection of rabbits

Groups of six female New Zealand white rabbits (Charles River Laboratories, 2.2–2.5 kg) were infected with the wild-type or mutant Vollum strains. Since deletion of the toxins abolishes the ability of the mutant to effectively establish an infection when administered intranasally spore infection [6], we used a previously described [5] intravenous (IV) infection procedure for the mutant. VollumΔ*pag*Δ*lef*Δ*cya* spores were germinated by incubation in Terrific broth for 1 hr, and then incubated in DMEM-10% FBS for 2 hr, to induce the capsule formation. The capsule was visualized by negative staining with India ink. The encapsulated vegetative bacteria (a dose of 5x10^6^ CFU) were used to intravenously (IV) inoculate rabbits.

Intranasally (IN) infected rabbits were anesthetized by subcutaneous injection of 1.5 ml of a 1:2 mixture of xylazine (20 mg/ml) and ketamine (100 mg/ml), and consequently infected with Vollum spores. Prior to infecting the animals, spore preparations were heat-shocked (70°C, 20 min) and serially diluted in saline to produce spore suspensions of 10^6^ CFU/ml. A dose of 1 ml was administered IN to each animal. The animals were monitored at least once a day for clinical signs that included changes in behavior such as response to environmental stimuli such as noise, as well as food consumption and signs of respiratory distress. All the histological analyses were performed on preserved organs, from animals who succumbed to the infection 48-72hrs post infection. Common state of the art practices in the field of anthrax study do not include the use of potent analgesics, as they affect disease progression and time to death. Death from anthrax was confirmed by post mortem plating of blood samples. Animals were euthanized when one of the following symptoms was detected: severe respiratory distress or the loss of righting reflex. Rabbits were sacrificed by sodium pentobarbitone injection. In the kinetics experiment, the rabbits (n = 6) were infected IN with Vollum spores and sacrificed 30 hr post infection, prior to the development of significant clinical symptoms.

This study was carried out in strict accordance with the recommendations of the Guide for the Care and Use of Laboratory Animals of the National Research Council and the safety guidelines of handling tier 1 agents. The protocols were approved by the IIBR Institutional Animal Care and Use Committee (permit numbers RB-25-2013 and RB-11-16).

### Tissue processing for histopathology

Brains chosen for histological analysis were harvested from rabbits that succumbed to the infection, or at designated time points (controls). The brains were immediately placed in 50ml tubes containing ~30 ml of 3.7% formaldehyde in PBS for fixation. After fixation, brains were cross-sectioned into 4–5 mm thick slices, each placed in a separate histological cassette, and the slices were paraffinized overnight in a Leica APS200 system (Leica Biosystems, Wetzler, Germany). The tissue slices were then embedded in paraffin blocks. Consequently slides were prepared by mounting 5μm thick sections prepared using a rotary microtome (Leica Biosystems, Wetzler, Germany)

### Histopathological staining

Prepared sections were subjected to either H&E staining (using a protocol modified for brain staining with a prolonged hematoxylin staining step [15min]) or immunofluorescence staining for bacteria.

The immunofluorescence protocol included deparaffinizaton, staining with a polyclonal rabbit (Rb) serum against formalin-killed vegetative capsular bacteria (reagent produced in-house) as the primary antibody and a commercial donkey-anti-rabbit-Atto-594 antibody combined with DAPI as the secondary staining phase (Sigma). Coverslips were mounted with Fluoromount (Sigma).

### Image acquisition

H&Images were acquired using a Zeiss Axioskop microscope (Zeiss, Oberkochen, Germany) equipped with a Nikon DS-Ri1 camera controlled by a DS-U3 Digital Sight and the Nis-Elements-Br software suite (Nikon, Tokyo, Japan). Fluorescent images were acquired using a Zeiss LSM 710 confocal microscopy system (Zeiss, Oberkochen, Germany)

## Results

**Fig 1:**
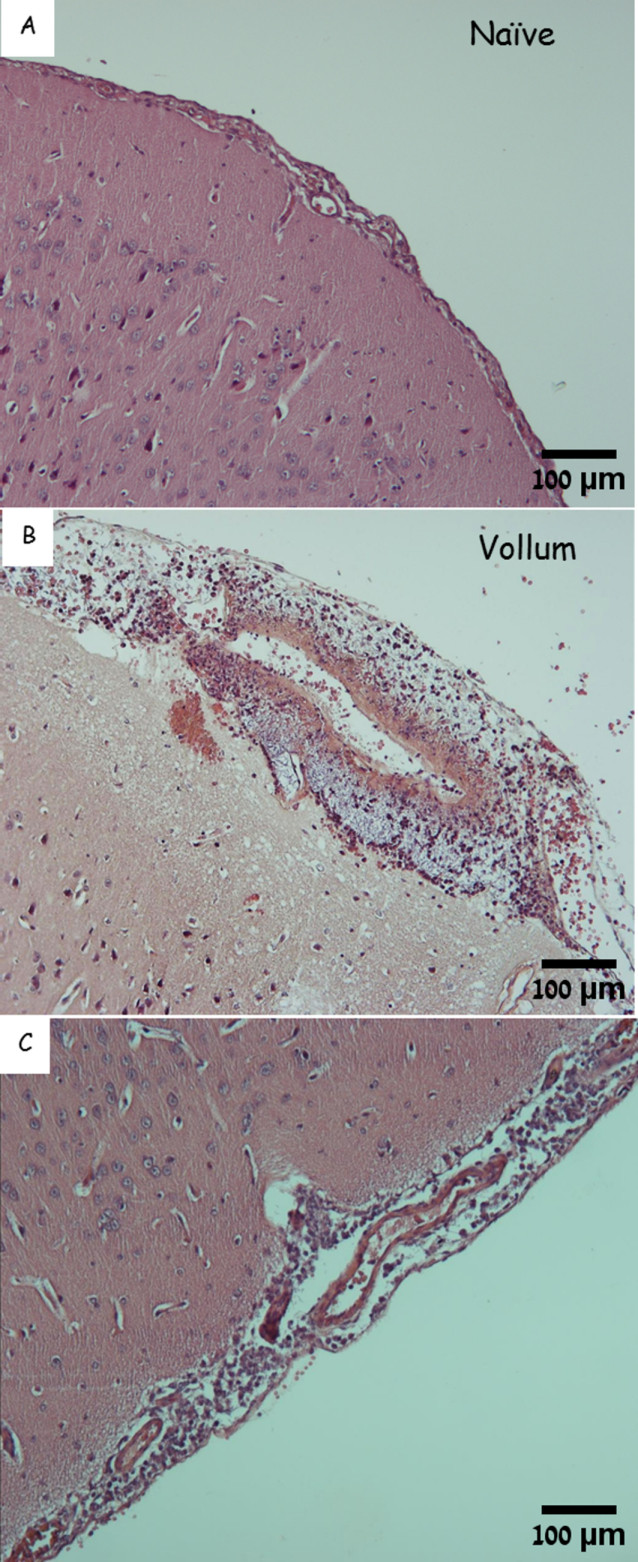
The meninges of naïve and infected rabbits. H&E staining of the cortex and meninges from a naïve rabbit (A), from representative rabbits that succumbed to IN instillation of Vollum spores (B), or to IV inoculation of VollumΔTox bacteria (C). Magnification–x100. While meninges of the naïve brain are tight around the parenchyma (A), the meninges from the Vollum infected animals are dilated and filled with bacteria and erythrocytes (B). In close proximity to the meninges, parenchymal bleeding could be detected (B). The meninges of the toxin-null strain are rich with bacteria and immune cells (C).

During the systemic phase of anthrax vegetative bacteria disseminate via the bloodstream throughout the host body and accumulates in various tissues, including the brain. The major histopathological finding is meningitis. We found massive bacterial growth in the meninges (Fig 1B) but no evidence of encephalitis, even when the bacteria seemed to emerge from parenchymal blood vessels into the parenchyma itself. Erythrocytes were present within the meningeal space but no clear vasculitis was detected.

As previously described, the finding of macroscopic brain hemorrhages post mortem was rare. When observed, hemorrhages typically tended to be found in the cerebellum. Histology of the meninges (Fig 1B) revealed regions of edema in the meninges with microscopic hemorrhages in parenchymal tissue. The meningeal edema contained high concentrations of bacilli, concurrent with significant infiltration of immune cells (mainly lymphocytes) and low concentrations of erythrocytes. Surprisingly, the blood vessels seemed intact with negligible signs of vasculitis. Similar edema, though significantly lower, could be found in rabbits that succumbed to IV inoculation of the toxin-deficient mutant (Fig 1C). In this case the immune response includes a much more massive infiltration of immune cells, mainly lymphocytes, but lacking detectable erythrocytes. This extensive immune response might indicate that the absence of the immunosuppressory toxins results in a heightened immune response. Despite this response, however, this infection develops, overcoming the fully activated immune system. The finding of bacilli and absence of clear vascular damage is typical to anthrax-meningitis.

**Fig 2:**
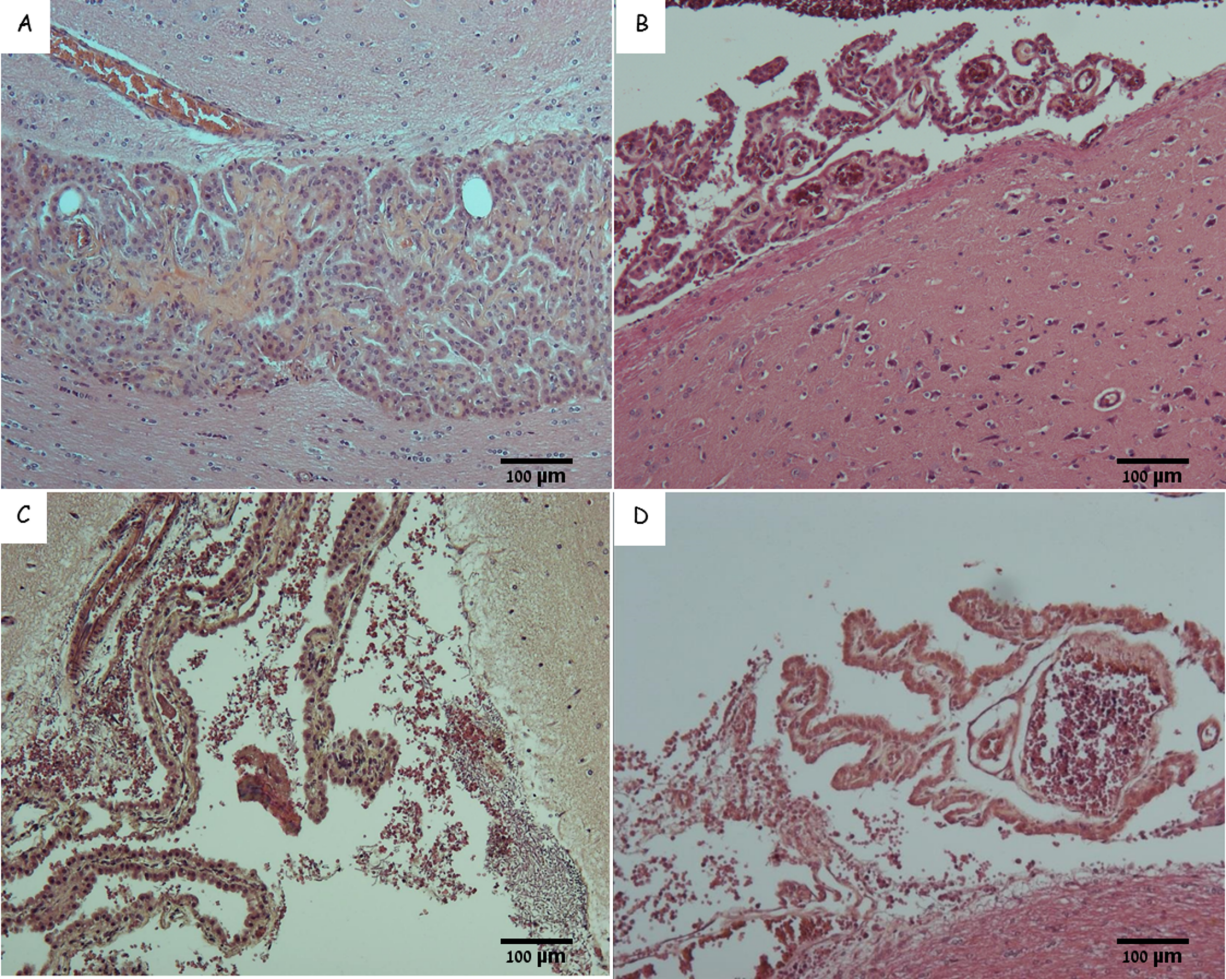
Choroid plexus of naïve and infected rabbits. H&E staining of choroid plexus from a naïve rabbit (A and B), from representative rabbits that succumbed to IN instillation of Vollum spores (C), or to IV inoculation of VollumΔTox bacteria (D). Magnification x100. The typical structure of the choroid plexus from a naïve brain (A, B) with normal size blood vessels (B), in contrast to the damaged structure containing bacterial clamps in the CSF of the Vollum (C) or VollumΔtox (D) infected animals.

Examination of the CP in rabbits that succumbed to IN spore infection revealed that while major parts were intact, other regions of the tissue were significantly damaged to the point of complete destruction (Fig 2C). We found massive destruction of entire sections of the CP coupled with massive aggregation of bacilli in the ventricles in close proximity to the parenchyma. The CP also contained large amounts of intra vascular bacterial aggregates, often enclosed in what appeared to be fibrin-like clots. Erythrocytes and bacilli were abundant in the vicinity of the damaged CP area, indicating massive hemorrhages into the CSF. Once again this process is not toxin dependent since similar findings were documented in rabbits that succumbed to IV inoculation with the toxin deficient mutant (Fig 2D). The lower concentration of Bacilli in the CSF can be correlative to the lower bacteremia in animals that were inoculated IV [5].

**Fig 3:**
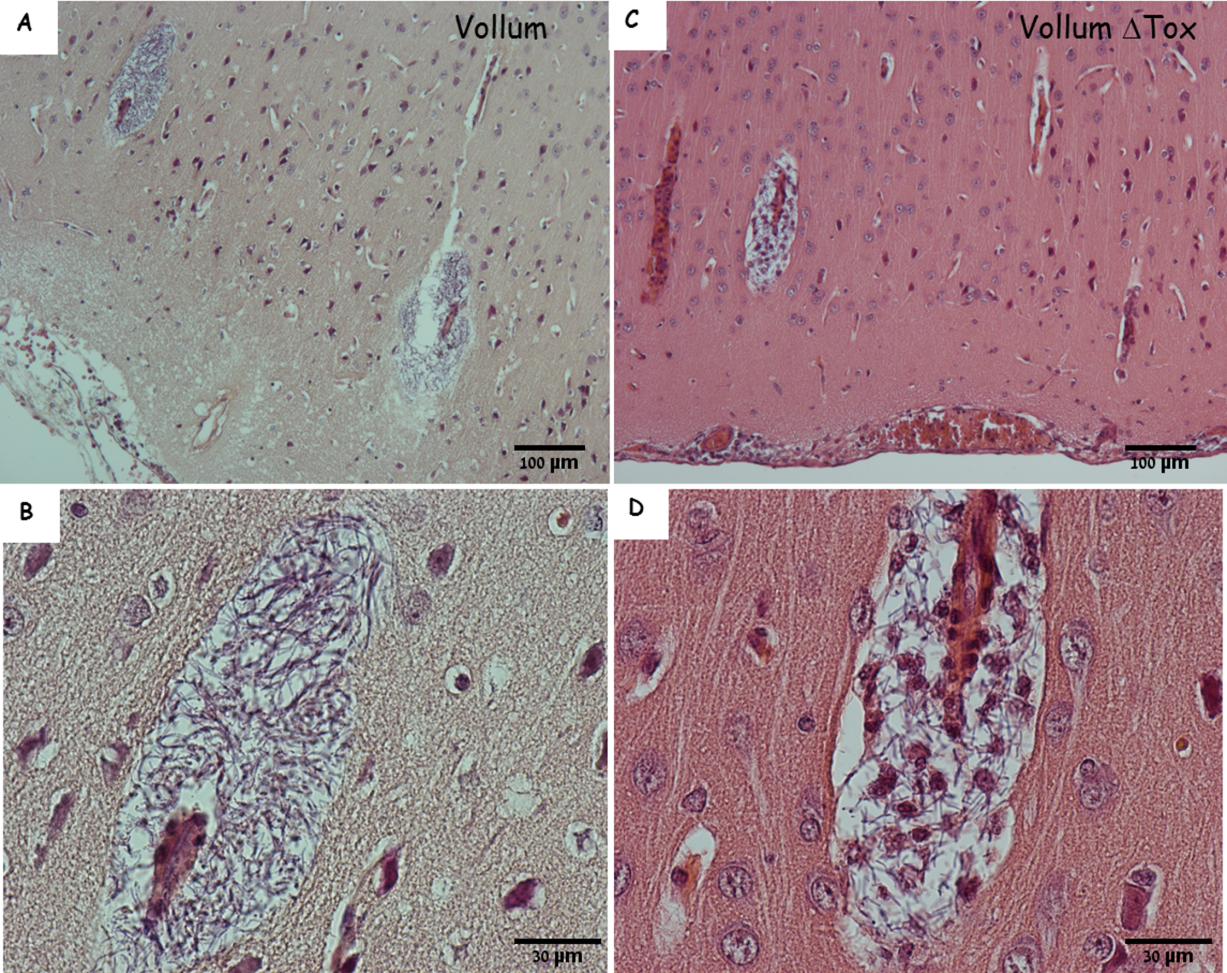
Paravascular bacterial growth in the cortex of infected rabbits. H&E staining of cortex and meninges from rabbits that succumbed to IN instillation of Vollum spores (A and B) or to IV inoculation of VollumΔTox (C and D). B and D are zoomed in into the relevant sectors of A and C. Magnification–A, C x100, B, D x400. Parenchymal bacterial growth in close proximity to the blood vessels. In the absence of toxins this bacterial growth is accompanied by immune cells (C, D). The growth is limited to extra-vascular space and not into the parenchyma.

In some cases, bacterial growth could be detected within what seem to be a paravascular space (Fig 3). This colonization could represent CSF-driven migration of bacteria from the point of entry into these extra-vascular spaces, resulting in the colonization of these spaces. Alternatively this growth could result from the penetration of bacteria from the blood into the brain via the blood brain barrier and represent an additional blood-brain crossing point. Unlike the CP, this type of BBB penetration requires specialized capacities such as endothelial attachment and penetration, but no significant adherence to the endothelium could be detected *in vivo* or *in vitro*. Once again this process is toxin independent since this phenomenon could be detected following i.v. inoculation of the toxin deficient strain (Fig 3C and 3D). The major difference between the toxin-producing and null mutant is the presence of immune cells in the immediate vicinity of the bacteria (Fig 3B and 3D). This finding correlates with the immunosuppressive role of the toxins [26]. This finding also demonstrates the robustness of *B*. *anthracis'* capability of colonizing the host even in the presence of a fully functional innate immune cellular response.

**Fig 4:**
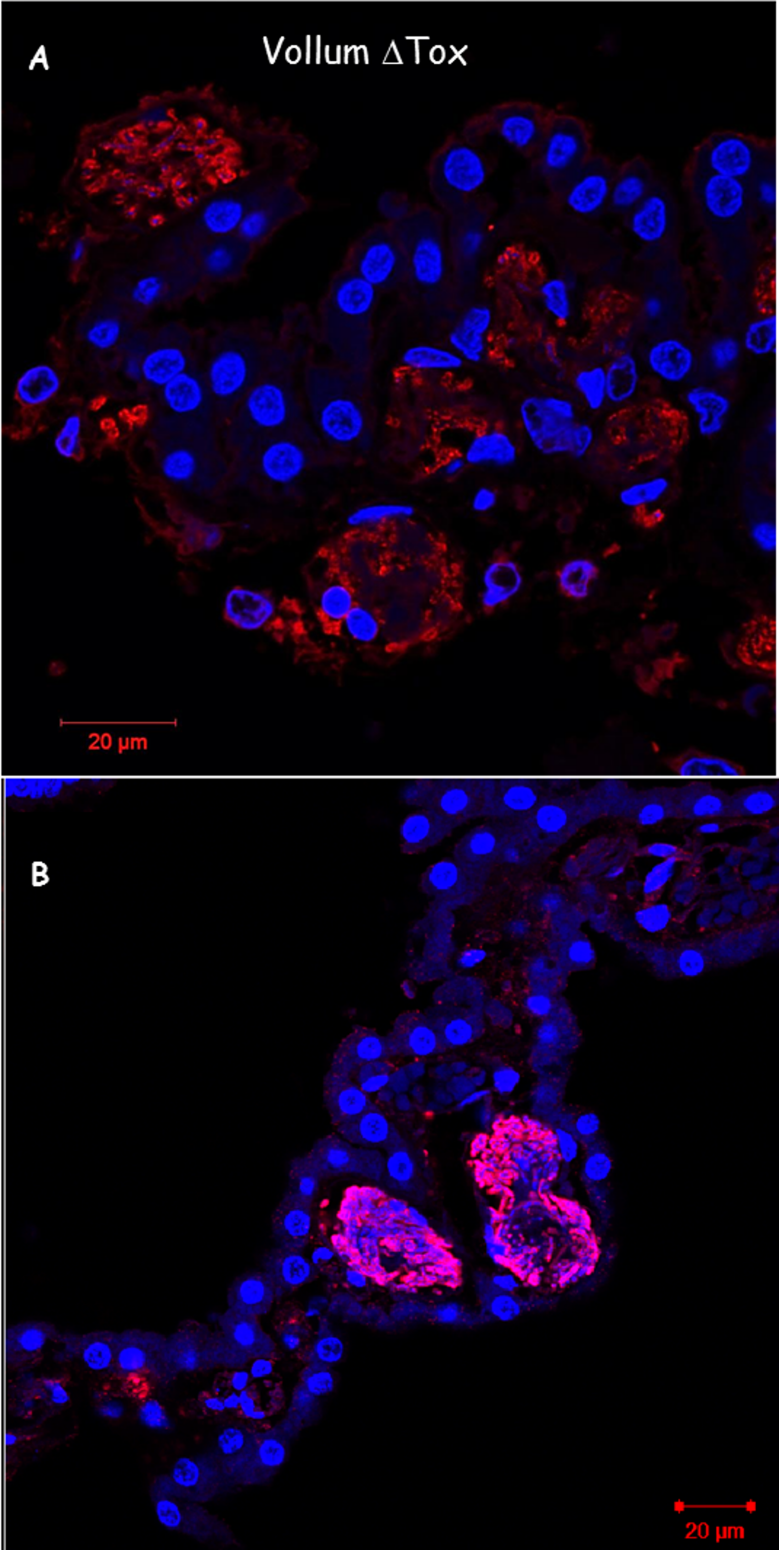
Immuno-histology of bacterial clumps in the choroid plexus. Immuno-histology with serum specific to the bacteria, of choroid plexus from rabbits that were inoculated IV with VollumΔTox bacteria. Red–bacteria, blue–DAPI stain of tissue nucleus. Magnification A, x600, B x400. Most of the bacteria are found in clusters at the periphery of the blood vessel.

**Fig 5:**
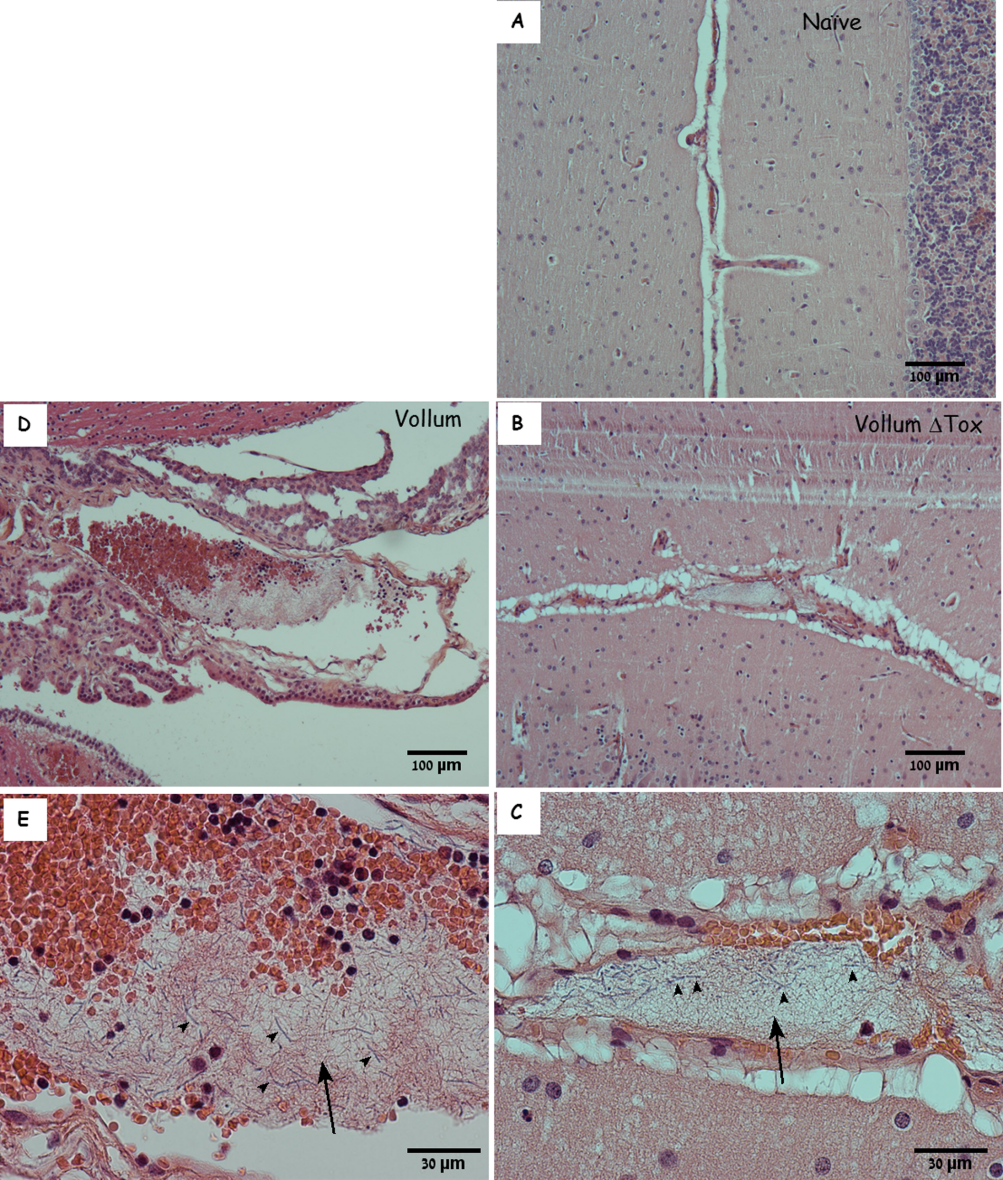
Bacteria-containing clots in brain blood vessels. H&E staining of rabbit cerebellum from naïve (A) and IV inoculated with VollumΔTox bacteria (B and C), and choroid plexus from IN spore inoculated (D and E) rabbits. The bacteria in dark blue (E and C) in reddish fibrin like matrix. Magnification A, B, C x100, C and E x600. Fibrin like structures are marked by arrows and bacteria by arrowheads.

**Fig 6:**
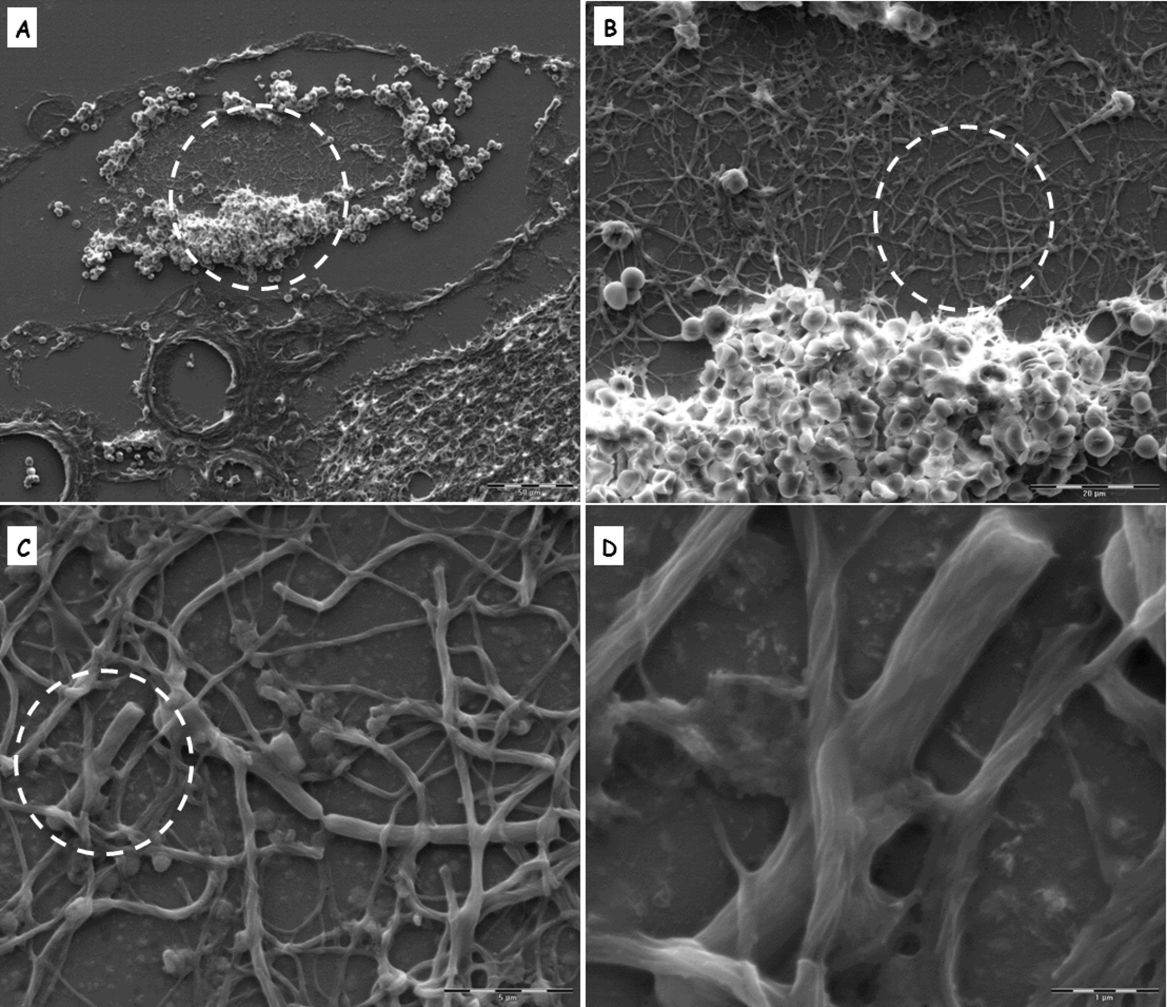
Scanning Electron Microscopy (SEM) of bacteria-containing clots. SEM analysis of blood vessel in the meninges with a clot (fibrin) trapping bacteria. Dashed circle indicates the area of the next magnification (increasing from A to D). The bacterium appeared to be trapped by fibers that resemble the typical structure of fibrin. The clot does not contain erythrocytes, cells, platelets or cell lysates (NET).

We used immunofluorescence techniques to label the bacteria in brain sections from animals that succumbed to infection with Vollum ΔTox strain. The results in Fig 4 demonstrate bacterial clusters in the CP. These bacterial clumps are organized as a ring along the vicinity of the blood vessel. H&E stain reveals complex structures composed of net-like aggregates entrapping/engulfing the bacterial cells (Fig 5). These structures could be found in large blood vessels in the CP (Figs 3, 5D and 5E), the cerebellum (Fig 5B and 5C) and the meninges (Fig 6). Such "bacterial clumps" were previously reported in lung and kidney, similarly located in major blood vessels [21, 22]. These structures may represent blood clots, though they lack the typical dense structures enriched with platelets and erythrocytes. Though similar structures are usually correlated with fibrin coagulation, we could not demonstrate significant fibrin staining by common fibrin staining methods, chemical or immunological. Histochemical staining with Phosphotungstic Acid Hematoxylin (PTAH) produced weak and inconclusive evidence of fibrin and collagen fibers. The collagen was present mainly in blood vessels in the meninges and could result from damage to the endothelial layer. Scanning Electron Microscopy (SEM) of these clots in meningeal blood vessels (Fig 6) revealed a filament-like structure that resembles that of fibrin [27, 28]. These filamentous structures form a fishnet-like structure across most of the blood vessel space. Other than bacteria, no additional cell types, immune or hematologic, are associated with this structure. At the periphery, a more typical clot structure was observed (Fig 6A and 6B).

**Fig 7:**
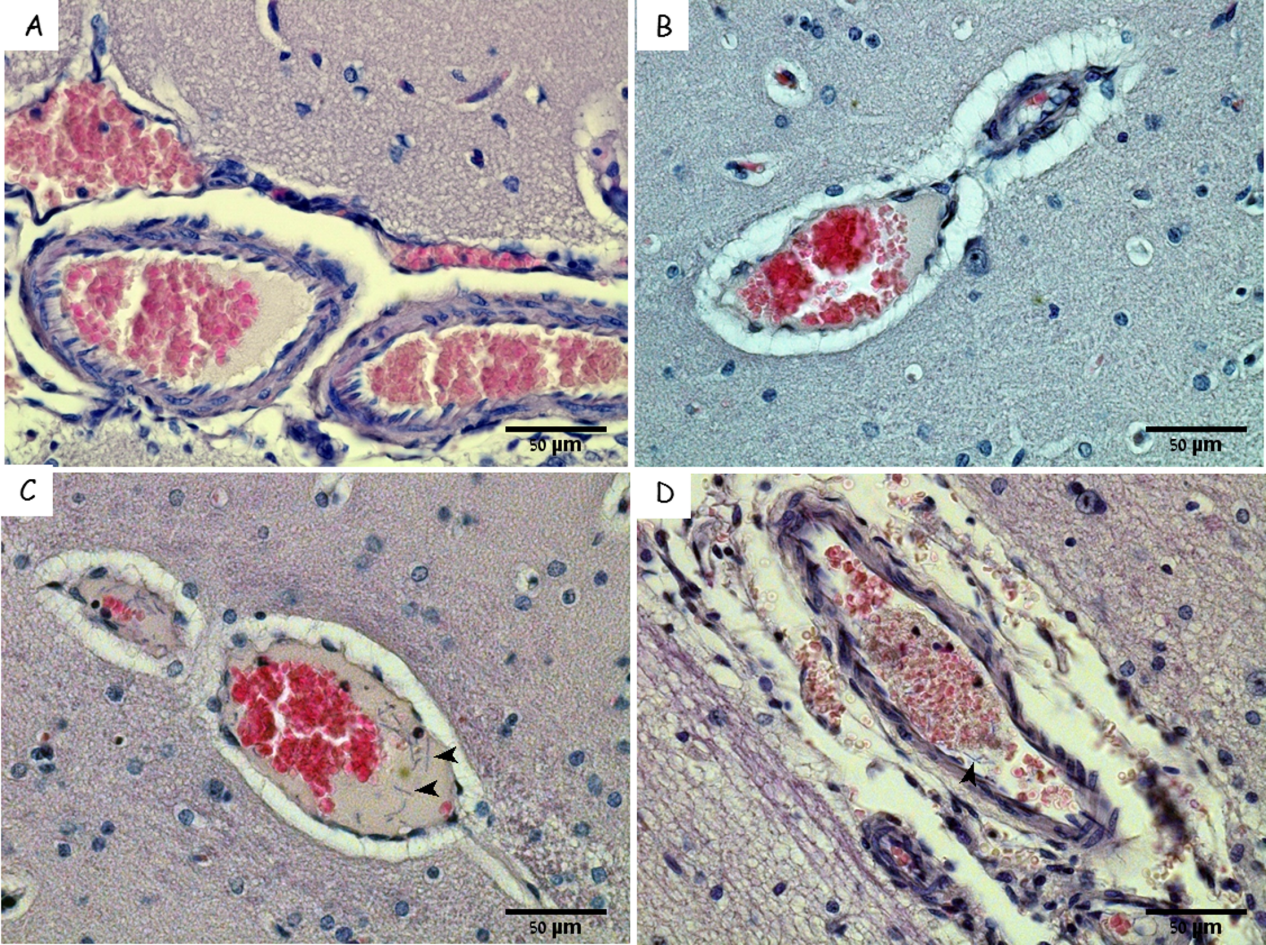
**Kinetic of bacteria-containing clots in brain blood vessels:** H&E staining of brains from IN infected rabbits at different stages of the disease A. Bacteremia of 1x10^5^ CFU/ml. B. Bacteremia of 2x10^6^ CFU/ml. C and D. Bacteremia of 1x10^8^ CFU/ml, two sites of the same brain. The accumulation of clots starts at bacteremia levels of 1x10^5^. Arrowheads marks bacteria in the clots.

To conclude, we compared the overall histological picture of brain infection as function of the bacterial load (Table 1). As can be seen in Fig 7A, 7B, 7C and 7D, the formation of clots starts at bacteremia of 10^5^ and clotting progresses as the bacterial load rises, with free bacteria appearing inside the vessels at higher concentrations.

**Table 1:** Arbitrary scoring of histopathological finding following infection with the wild type or toxin deficient mutant (relative to naïve pathology).

Finding	Vollum	VollumΔTox	Figure
**Meninges**			
Edema (Swelling)	+++	+	1
Bacterial growth	+++	++	1
Hemorrhage	++	-	1
PMN and Monocyte infiltration	++	+++	1
**Choroid plexus**			
Intravascular bacterial clamping	+++	+++	2, 4
Structure disruption	+++	+++	2
Bacteria clumps in CSF	+++	+++	2
**Para-vascular space**			
Bacterial growth	+++	+++	3
PMN and Monocytes infiltration	-	+++	
**Vascular**			
Edema (Swelling)	+++	+++	5
Fibril like structures	+++	+++	5,6
Bacteria trapped in structures	+++	+++	4, 5,6

- ≤10%, + 25%, ++ 50%, +++ ≥75%

## Discussion

Meningitis in anthrax is considered a complication of the systemic disease mainly in humans and NHP, with major negative prognostic implications. We and others have previously demonstrated meningitis pathology in guinea pigs and rabbits [12, 15, 20–23]. In fact, we identify meningitis in all animals that succumbed to *B*. *anthracis* infection, in both the guinea pigs and rabbit models. However, *B*. *anthracis* is not a classical central nervous system pathogen. The encapsulated bacterium adheres poorly to endothelial cells *in vitro* and close interactions between the bacteria and the endothelium *in vivo* are rarely documented. Published data of *in vitro* experiments utilizing tissue cultures demonstrate that BslA plays a major role in the adherence of the non-capsulated ΔpXO2 *B*. *anthracis* strain [19]. This BslA dependent adherence was shown to plays a minor role in the capsulated- bacteria attachment to the endothelium, probably due to physical interference by the capsule.

As can be clearly seen in Fig 6 and Fig 7, the formation of clots is a dominant feature in the development of the disease. Besides the well-known hemodynamic roles of blood coagulation, it was demonstrated that coagulation acts as part of the immune response [29]. In lower organisms, such as the horseshoe crab, blood coagulation is the only immune response against bacterial invasion [29]. Disseminated intravascular coagulation (DIC) is triggered in response to sepsis [30] and is a well-documented response to several gram negative infections [31]. Nevertheless, DIC usually causes non-specific coagulations in small blood vessels, while our findings demonstrate that this anthrax-induced filamentous structure is present mainly in large blood vessels. Gram positive bacteria such as *Staphylococcus aureus* and *Streptococcus pyogenes* developed specific pathways for inducing or evading blood clots as a crucial part of their pathogenesis. In *S*. *aureus* [32], three essential secreted virulence factors, Coa, vWbp and ClfA, were reported to be involved in coagulation. In Streptococcus group A (*S*. *pyogenes*), M protein was shown to induce coagulation, while streptokinase was found to be essential for releasing the bacteria from fibrin clots. Both protein are essential for pathogenesis [33]. We could not find homologs of the M protein or streptokinase in *B*. *anthracis*. However, open reading frames with homology to vWFA and fibrinogen binding protein A are present and might take a part in these processes.

SEM analysis of the filamenteous structures discussed herein (Fig 6C and 6D) showed that they are similar, but not identical, to those previously described in *in vitro* plasma coagulation [28] induced by *S*. *aureus* [32] and *S*. *pyogenes* [33]. The disparities between the structures observed in this work and previous data described above may result from the difference between *in vitro* and *in vivo* coagulation, or by the different trigger for coagulation. It is also possible (though unlikely) that these fibers are not fibrin, but bacterial-originated polymers and that the observed fibrous-structure is actually a bio-film. The possibility that these clots are neutrophil extracellular traps (NETs) was rejected since specific NET immune-staining was negative and since SEM analysis (Fig 6) detected none of the cellular debris or other typical NET structure-components.

**Fig 8:**
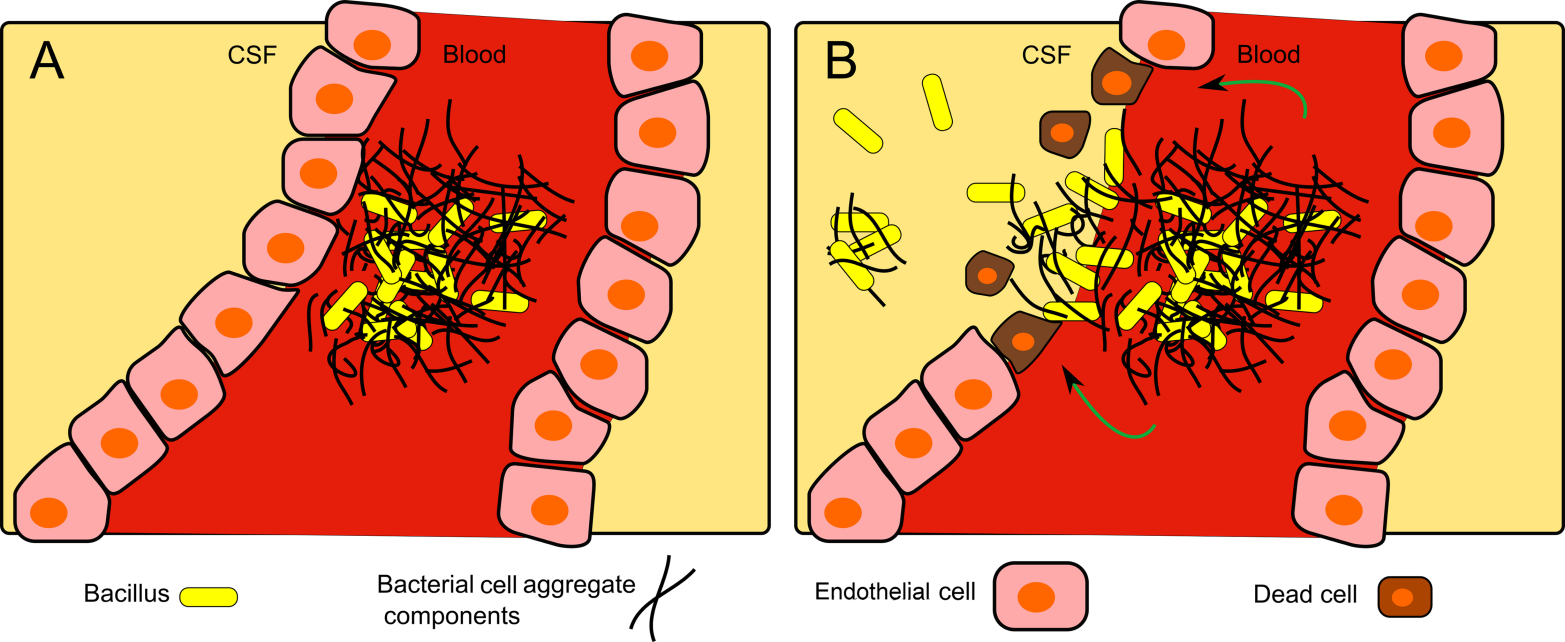
**Illustration of proposed aggregate-based CSF infiltration model:** A. Bacteria form aggregates in fibrin-like structures inside blood vessels. B. From within the aggregate, bacterial proteins damage the endothelial cells, causing vessel rupture, allowing the bacterial crossing of the blood brain barrier.

The choroid plexus (CP) appears to be the point of CNS entry. The high density of bacterial aggregates (Figs 4, 5D and 5E) in the CP and the extensive damage to this tissue (Fig 2C and 2D) might be explained as the result of secreted bacterial proteins/enzymes within the clot, in combination with the resulting increased local blood pressure, which ultimately lead to the rupture of the blood vessels and bacterial leakage into the CSF and the meninges (Fig 8).

All these processes are toxin-independent, since similar findings were observed in animals that succumbed to the toxin deficient mutant compared to those who were infected with the toxin-producing wild type strain. Given that the major role of the lethal and edema toxins is interference with the effective immune response, our results show that bacterial CNS invasion can occur in the presence of an active innate immune response. This fact is evident in Fig 1C and Fig 3D where massive infiltration of immune cells can be seen in intimate vicinity to the bacteria. Indeed, this infiltration was even more evident in the brains of animals infected with the toxin-null mutant. Despite the prevalence of the immune cells and the lack of inhibitory toxins, the invading immune cells failed to protect the animals from CNS infection. In fact, this infiltration by itself could damage the blood vessel compromising the BBB. Nevertheless, this infiltration was free of erythrocytes, indicating that the damage to the vascular system was not severe enough to result in hemorrhages.

Although no major vasculitis could be determined in the meninges or the parenchyma, bacterial growth could be detected in the meningeal space and surrounding blood vessels within the parenchyma (Figs 1 and 3). In addition, the presence of erythrocytes indicated hemorrhaging that probably originated from the ruptured choroid plexus or damaged blood vessels in the meninges. The bacterial growth surrounding blood vessels in the parenchyma, on the other hand, appeared to be clear of erythrocytes (Fig 3B). Though it appears to contain only bacteria, the growing bacteria could also be forming a fibrin-like structure, similar to that observed in the blood vessel. Alternatively, this extra-venal space might be connected to the CSF circulatory tracts and to CSF-borne bacteria that reached this location and locally propagated. In the presence of an active immune system (the absence of toxins) this growth attracted immune cells from the circulation.

Quantitative pathology of inhalational anthrax patients from the Sverdlovsk incident [13] revealed meninges containing *B*. *anthracis* in 23 of the 29 cased that were analyzed (79%) and low or high levels hemorrhage in practically all cases. Meningeal and parenchymal fibrin was reported in 83–90% of cases. The documented cases of meningitis presented similar findings to those we showed in the rabbit model (Fig 1B), demonstrating Bacilli, erythrocytes and edema in the subarachnoid space [13]. The perivascular *B*. *anthracis* accumulation, which appeared similar to that depicted in Fig 3, was reported as occupying the Virchow-Robin spaces in the cerebral cortex [13]. We therefore conclude that the bacilli accumulation described in Fig 3 likewise occupies the Virchow-Robin spaces. The Virchow-Robin spaces are invasions of arteries from the subarachnoid space into the cortex, surrounded by CSF and pia mater. In direct contact with the meninges and separated from the parenchyma by pia mater, these areas may be the source of the bacteria and immune cells. Therefore, this pathology represents meningitis rather than encephalitis. In addition, these findings suggest that the bacteria hypothetically originate from the choroid plexus, into the CSF and the meninges, branching from there into the Virchow-Robin spaces. In this case we predict that the ultimate cause of death could be increased intracranial pressure and subsequently, a shutdown of the respiratory center in the Medulla oblongata (brain stem).

During the late stages of the disease, the role of toxins seems redundant, as the overall pathology of these processes is similar regardless of the toxins' presence. Nevertheless, two major differences could be detected between toxinogenic and toxin-deficient strains (Table 1): **A.** Signs of hemorrhage in the parenchyma could be detected only in the presence of toxins. This finding resembles the findings in humans [13] and NHP [12, 23], though the scope is lesser in the animal models described here. The difference in symptoms severity could result from longer duration of anthrax in humans (a minimum 6 days) [13] compared to only 2 to 4 days in rabbits. **B.** As expected, in the presence of the immunosuppressive activity of the toxins, markedly lower levels of immune cells (mainly lymphocytes) could be detected infiltrating the meninges. However, in the absence of toxins, the subarachnoid space is heavily infiltrated with polymorphonuclear cells (Table 1, Figs 1 and 3). While this may indicate a somewhat different disease course, the final outcome is very similar. This finding also has implications on the possible efficacy of toxin neutralizing therapeutics during the advanced stages of anthrax, since the target of such drugs is redundant at this stage, probably resulting in minor effects on the development of meningitis.

In conclusion, we propose that the first step of anthrax-meningitis is bacterial adhesion to the blood vessel by usurping blood coagulation, mainly in the choroid plexus. The trapped bacteria then destroy sections of the choroid plexus, resulting in penetration into the CSF and leading to meningitis and hemorrhage (Fig 8). Death could be the result of increased intracranial pressure and/or damage to the brain stem.

## Supporting information

S1 ARRIVE (PDF)
